# The Association Between Fermented Food Intake and Hs-CRP Across Age Groups in Korean Adults: Effect Modification by Sodium Intake

**DOI:** 10.3390/nu18081264

**Published:** 2026-04-16

**Authors:** Woori Na, Cheongmin Sohn

**Affiliations:** 1 Institute of Life Science and Natural Resources, Wonkwang University, 460 Iksandaero, Iksan, Jeonbuk 54538, Republic of Korea; nawoori6@gmail.com; 2 Department of Food and Nutrition, Wonkwang University, Iksan, Jeonbuk 54538, Republic of Korea; 3 Department of FoodTech, Jeonbuk Advanced Bio-Convergence Academy, Wonkwang University, 460 Iksan-daero, Iksan, Jeonbuk 54538, Republic of Korea

**Keywords:** fermented foods, *C*-reactive protein, sodium, inflammation, Korea

## Abstract

**Background/Objectives**: Korean traditional fermented foods may confer metabolic and anti-inflammatory benefits; however, their high sodium content raises concerns, particularly given age-related differences in sodium sensitivity. This study examined age-specific associations between fermented food intake and high-sensitivity *C*-reactive protein (hs-CRP), a marker of low-grade systemic inflammation, and assessed whether sodium intake modifies these associations. **Methods**: Data from KNHANES 2015–2018 were used to analyze 17,984 adults. Fermented foods were categorized into 10 groups (grains, jang, vinegars, vegetables, fish, fruits, dairy, alcoholic beverages, sauces, and tea/beverages). Intake (% of total energy) was classified into quartiles. Elevated hs-CRP was defined as ≥1 mg/L. Complex-sample multivariable logistic regression was used to assess age-stratified associations and interactions with total sodium and fermented food-derived sodium (SPSS 29.0; *p* < 0.05). **Results**: Fermented food intake decreased with age (*p* < 0.001). In adults aged 20–39, higher intake was associated with lower odds of elevated hs-CRP (Q4 vs. Q1: OR = 0.699, 95% CI 0.542–0.901; p for trend = 0.002). A similar inverse association was observed in those aged 40–64 (Q4: OR = 0.817, 95% CI 0.691–0.967; p for trend = 0.006), which remained significant after adjustment for fermented food-derived sodium. Among adults ≥65, significant interactions were observed for both fermented food-derived sodium (*p* = 0.040) and total sodium (*p* = 0.042), indicating variation across sodium intake levels. **Conclusions**: The association between fermented food intake and systemic inflammation differs by age. In older adults, this relationship appears to be modified by dietary sodium context, highlighting the importance of age-specific dietary considerations.

## 1. Introduction

Chronic low-grade inflammation is increasingly recognized as a central mechanism in the development and progression of cardiovascular and metabolic diseases [1], with dietary factors identified as among the most influential modifiable determinants of inflammatory status [2,3]. High-sensitivity *C*-reactive protein (hs-CRP) serves as a widely adopted marker of systemic inflammation, with demonstrated prognostic value for cardiovascular events and all-cause mortality across a broad range of concentrations. Identifying dietary factors associated with hs-CRP levels, therefore, carries direct relevance for chronic disease prevention strategies. Among dietary components, sodium has received growing attention beyond its well-established role in blood pressure regulation. Both experimental and observational evidence suggest that excessive sodium intake promotes systemic inflammation through immune cell priming and vascular endothelial impairment, with downstream implications for chronic disease risk [4,5,6,7]. Of particular relevance, physiological sensitivity to sodium appears to increase with advancing age, a pattern attributable to declining renal sodium clearance, altered vascular reactivity, and diminished immune regulation in older individuals [8,9]. Whether the level of sodium intake influences the relationship between dietary exposures and inflammatory outcomes across age groups remains insufficiently examined.

Fermented foods have garnered research interest as a dietary source with potential relevance to inflammatory regulation. Through the production of organic acids, bioactive peptides, and microbial metabolites, fermentation may favorably alter the gut environment and modulate host immune function, and these foods are widely considered to confer health benefits [10]. Several experimental studies have reported inverse associations between fermented food intake and inflammatory markers, though the bulk of this evidence is drawn from trials focusing on discrete products, namely fermented dairy, fermented soy, and kimchi [11,12,13]. The findings across these studies have not been consistent [14], and research assessing fermented food consumption at a broader dietary level remains sparse. Such inconsistency raises the possibility that the anti-inflammatory effects of fermented foods are not uniformly expressed, and that unexamined contextual factors may account for this variability.

The Korean dietary context provides a relevant case for examining this relationship. Fermented food consumption in Korea is predominantly centered on kimchi and jang-based products, which together constitute a substantial share of daily energy intake [15]. Older and middle-aged adults, who are more likely to adhere to home-prepared traditional eating practices, have been reported to rely more heavily on these foods relative to younger generations [16]. Notably, the fermented foods that dominate the Korean diet are also among its primary sources of dietary sodium, a characteristic that distinguishes the Korean context from Western dietary settings, where fermented foods tend to be lower in sodium, consisting largely of dairy and fermented vegetables [17,18]. This structural coupling between fermented food consumption and sodium load raises the possibility that sodium intake may serve as a meaningful contextual factor shaping the inflammatory implications of fermented food consumption in this population.

The extent to which the fermented food–inflammation relationship is contingent on sodium intake, and whether this dynamic varies across age groups, has received limited empirical attention. Prior research has generally examined fermented food consumption as a single exposure, with limited consideration of the potential modifying role of sodium and age-related variation in its effects. The present study, therefore, examined the association between fermented food intake and hs-CRP across age groups in Korean adults, and investigated whether sodium intake level moderated this relationship.

## 2. Materials and Methods

### 2.1. Study Population

**Figure 1:**
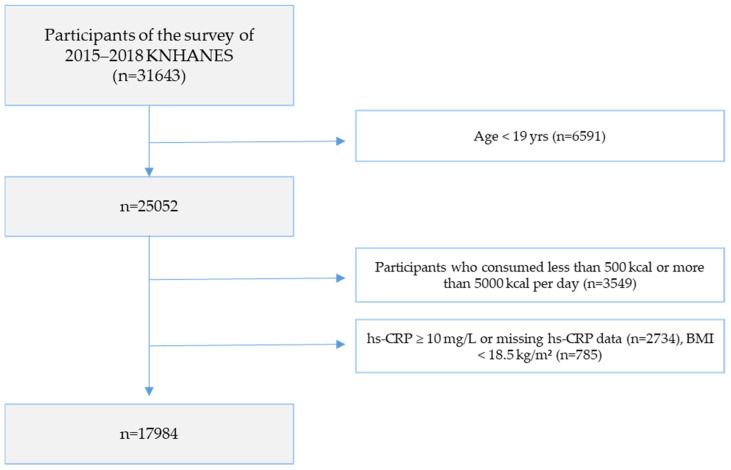
Flowchart of participant selection.

This study utilized raw data from the 6th and 7th Korea National Health and Nutrition Examination Survey (KNHANES; 2015–2018), provided by the Korea Disease Control and Prevention Agency (KDCA). Of the 31,649 participants in the survey, adults aged 19 years and older were initially selected (*n* = 25,052). Participants were subsequently excluded based on the following criteria: those with a daily total energy intake of less than 500 kcal or more than 5000 kcal (*n* = 3549); those with missing hs-CRP values or hs-CRP levels of 10 mg/L or higher (*n* = 2734), and those with BMI < 18.5 kg/m^2^ (*n* = 785). The final analytic sample comprised 17,984 individuals (Figure 1). This study was conducted after receiving approval from the Institutional Review Board of Wonkwang University (Approval No. WKIRB-202412-SB-088).

### 2.2. Fermented Food Classification

Fermented food groups were classified based on previous international studies [19,20,21,22,23]. In a prior study by our research group, On et al. [24] adapted these international classification frameworks to reflect the dietary and cultural characteristics of the Korean population, establishing a ten-group classification: grains, fermented soybean products, vinegar, vegetables, fish and seafood, fruits, dairy products, alcoholic beverages, sauces, and leaf teas and beverages. The present study adopted this Korean-adapted classification without modification.

The grain group comprised fermented grain-based products, including fermented breads and jeungpyeon (steamed rice cake leavened through fermentation). The jang group included fermented soybean-based condiments, namely soy sauce, doenjang (fermented soybean paste), gochujang (fermented red pepper paste), cheonggukjang (fast-fermented soybean paste), ssamjang (seasoned fermented paste), and chunjang (black bean paste). The vinegar group encompassed plain vinegar and vinegar-based beverages. The vegetable group covered kimchi and other pickled vegetables, including danmuji (yellow pickled radish) and jangajji (vegetables pickled in soy sauce or brine). The fish group comprised jeotgal (salted fermented seafood), aekjeot (fermented fish sauce), and other fermented fish products. The fruit group included fermented fruit-based products such as olives. The dairy group consisted of fermented milk products and cheese. The alcoholic beverage group included fermented beverages such as beer, wine, makgeolli (rice wine), fruit wine, and cheongju (clear rice wine). The sauce group covered fermented condiments including balsamic dressing and hot sauce, and the tea·beverage group consisted of fermented teas.

### 2.3. Covariate

General and health-related characteristics of the study participants were obtained from health examination data, including sex, age, body mass index (BMI), waist circumference (WC), household income level, education level, marital status, hypertension, diabetes, cardiovascular disease, cancer diagnosis, frequency of binge drinking, smoking status, and aerobic physical activity. BMI was calculated and reported to two decimal places. Household income level was categorized into four groups: ‘low’, ‘middle-low’, ‘middle-high’, and ‘high’; education level was similarly divided into four groups: ‘elementary school or below’, ‘middle school’, ‘high school’, and ‘college or above’. Marital status was categorized as currently married or single. Alcohol consumption, smoking status, and aerobic physical activity were each reclassified into binary variables representing current drinking status (current drinker vs. non-drinker), smoking experience (ever smoker vs. never smoker), and current aerobic physical activity status (active vs. inactive), respectively. Hypertension, diabetes, cardiovascular disease, and cancer diagnosis were defined as binary variables (yes or no) based on physician diagnosis. Cardiovascular disease was defined as a physician diagnosis of at least one of the following: angina pectoris, myocardial infarction, or stroke. Cancer diagnosis was defined as having been diagnosed with at least one of the following: gastric, liver, colorectal, breast, cervical, lung, or thyroid cancer.

### 2.4. Statistical Analysis

All analyses accounted for the complex sampling design of KNHANES, consistent with the cross-sectional nature of the study. Participants were stratified into three age groups: 20–39 years, 40–64 years, and 65 years and older. Baseline characteristics were compared across age groups, with continuous variables presented as mean ± standard error and categorical variables as frequency and weighted percentage. Between-group differences were assessed using the complex samples general linear model for continuous variables and the Rao-Scott chi-square test for categorical variables. hs-CRP was dichotomized at 1 mg/L, the lower boundary of the moderate-risk category in the AHA/CDC cardiovascular risk classification, which has been applied as a sensitive cut-point for detecting low-grade chronic inflammation in general adult populations [25,26]. Fermented food intake was energy-adjusted and categorized into quartiles (Q1–Q4) for all regression analyses. Age-stratified multivariable logistic regression was conducted to examine the association between fermented food consumption and elevated hs-CRP, with results expressed as odds ratios (ORs) and 95% confidence intervals (CIs). Covariates were selected based on prior literature and univariable screening. Model 1 was adjusted for sex, age, cardiovascular disease, cancer diagnosis, WC, and total energy intake. Model 2 was further adjusted for total sodium intake (g/day) in addition to Model 1 covariates. Model 3 was further adjusted for fermented food-derived sodium intake (g/day) in addition to Model 1 covariates. The potential moderating role of sodium intake was assessed by incorporating a fermented food intake × sodium intake interaction term into the logistic regression model, with statistical significance evaluated using the p for interaction. To illustrate how the association between fermented food intake and hs-CRP varied across sodium intake levels, predicted probabilities of hs-CRP ≥1 mg/L were estimated for Q4 and Q1 at each sodium intake interval using logistic regression coefficients. The difference in predicted probability between quartiles (ΔP = P(hs-CRP ≥ 1|Q4) − P(hs-CRP ≥ 1|Q1)) was plotted with 95% confidence intervals, stratified by age group. All statistical analyses were performed using SPSS 29.0 (IBM Corp., Armonk, NY, USA). Statistical significance was defined as *p* < 0.05.

## 3. Results

### 3.1. General Information

Baseline characteristics of participants by age group are presented in Table 1. The distribution across age groups was as follows: 4410 individuals aged 20–39 years (45.1% men, 54.9% women), 8742 were aged 40–64 years (41.5% men, 58.5% women), and 4832 were aged 65 and older (43.8% men, 56.2% women). Mean BMI was 24.06 ± 0.066 kg/m^2^ in the 20–39-year group, 24.35 ± 0.041 kg/m^2^ in the 40–64-year group, and 24.37 ± 0.049 kg/m^2^ in the 65 years and older group, with significant differences observed across age groups (*p* < 0.001). Regarding household income, the proportion in the lowest income category was highest in the 65 years and older group (46.2%), whereas the highest income category was more prevalent in the 40–64-year and 20–39-year groups (37.1% and 33.4%, respectively, *p* < 0.001). Educational status also differed significantly across age groups; the proportion with elementary school education or below was highest in the 65 years and older group (58.1%), while college graduation or above was most common in the 20–39-year group (62.8%) (*p* < 0.001). With respect to lifestyle behaviors, the prevalence of binge drinking declined with advancing age (*p* < 0.001). The proportion meeting aerobic physical activity guidelines was highest in the 20–39-year group (55.1%) and decreased significantly with age (*p* < 0.001). The prevalence of chronic diseases increased markedly with age. Hypertension was present in 1.7%, 20.8%, and 55.7% of the 20–39-year group, 40–64-year group, and 65 years and older groups, respectively (*p* < 0.001), and diabetes prevalence followed a similar pattern at 0.5%, 7.7%, and 22.0% (*p* < 0.001). Mean hs-CRP levels were 0.265 ± 0.008 mg/L, 0.268 ± 0.005 mg/L, and 0.330 ± 0.008 mg/L in the 20–39-year, 40–64-year, and 65 years and older groups, respectively, increasing significantly with age (*p* < 0.001). The proportion of participants with hs-CRP ≥1 mg/L was highest in the 65 years and older group, with significant differences observed across age groups (*p* < 0.001).

### 3.2. Nutrient Intake and Proportion of Fermented Food Consumption by Age Group

**Figure 2:**
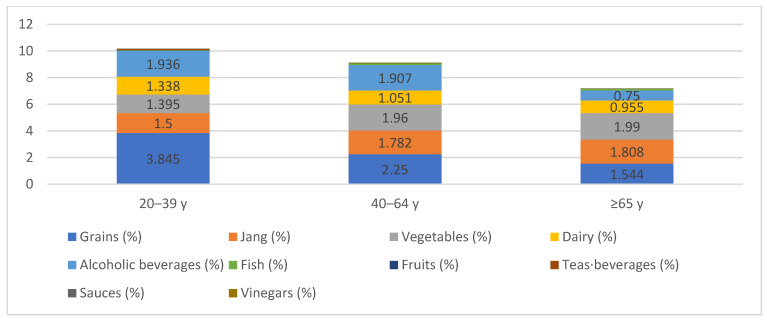
Distribution of fermented food intake by food group and age group. Values represent the mean percentage of total energy intake contributed by each fermented food group. All values were adjusted for total energy intake using the energy-adjustment method. Age groups are categorized as 20–39 years, 40–64 years, and ≥65 years.

Nutrient intake and the proportion of fermented food consumption by age group are presented in Table 2 and Figure 2. Total energy intake differed significantly across age groups, decreasing with advancing age from 2177.0 ± 15.3 kcal in the 20–39-year group to 2044.8 ± 10.3 kcal in the 40–64-year group and 1701.4 ± 11.6 kcal in the 65 years and older group (*p* < 0.001). Macronutrient intake differed significantly across age groups. Total energy, protein, fat, and sodium intake decreased with increasing age, whereas carbohydrate intake showed a relatively higher level in the middle-aged group. Dietary fiber intake was highest in the 40–64-year group (all *p* < 0.001).

The proportion of total energy derived from fermented foods decreased significantly with age, from 10.180 ± 0.183% in the 20–39-year group to 9.126 ± 0.114% in the 40–64-year group and 7.180 ± 0.126% in the 65 years and older group (*p* < 0.001). With respect to energy contribution and sodium load by fermented food group, the energy contribution of the vegetable group was 1.395%, 1.960%, and 1.990% in the 20–39 year, 40–64 year, and 65 year and older groups, respectively, with corresponding sodium contributions of 611.7 mg, 807.5 mg, and 742.7 mg (*p* < 0.001). The jang group contributed 1.500%, 1.782%, and 1.808% of total energy across the same age groups, with sodium contributions of 786.071 mg, 866.459 mg, and 796.403 mg, respectively (*p* < 0.001). Sodium derived from both the vegetable and jang groups was highest in the 65 and older group (Table 2B).

Fermented food group intake by age group is detailed in Supplementary Table S2. Intake from the grain and alcoholic beverage groups decreased significantly with advancing age, whereas intake from the vegetable and jang groups increased significantly (all *p* < 0.001). The dairy group showed statistically significant differences across age groups (*p* < 0.001). No significant differences were observed for the fruit, sauce, or tea·beverage groups.

### 3.3. Association Between Fermented Food Consumption and hs-CRP by Age Group, and the Moderating Effect of Sodium Intake

**Figure 3:**
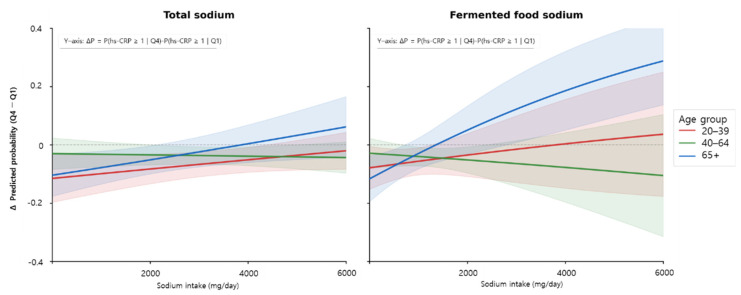
Difference in predicted probability of hs-CRP ≥1 mg/L between the highest (Q4) and lowest (Q1) quartiles of fermented food intake across sodium intake levels, stratified by age group. The y-axis represents ΔP = P(hs-CRP ≥ 1|Q4) − P(hs-CRP ≥ 1|Q1), indicating the difference in predicted probability of elevated hs-CRP between the highest and lowest quartiles of fermented food intake. Negative values indicate a lower predicted probability of hs-CRP ≥1 mg/L in Q4 relative to Q1, suggesting an anti-inflammatory association with higher fermented food intake. The left panel presents results adjusted for total sodium intake; the right panel presents results adjusted for fermented food-derived sodium intake. Shaded areas represent 95% confidence intervals. Analyses were stratified by age group: 20–39 years (red), 40–64 years (green), and ≥65 years (blue).

The association between fermented food intake and hs-CRP ≥1 mg/L by age group is presented in Table 3. In adults aged 20–39 years, higher fermented food intake was significantly associated with lower odds of elevated hs-CRP. Participants in the highest quartile (Q4) had significantly lower odds compared with those in the lowest quartile (Q1), and this association remained significant after sodium adjustment (Model 3: OR = 0.699, 95% CI: 0.542–0.901; p for trend = 0.002). No significant interaction with sodium intake was observed. In adults aged 40–64 years, an inverse association was observed, with significantly lower odds of elevated hs-CRP in Q4 compared with Q1 after full adjustment (Model 3: OR = 0.817, 95% CI: 0.691–0.967; p for trend = 0.006). In adults aged ≥65 years, no statistically significant direct association was observed between fermented food intake and hs-CRP. However, significant interactions with sodium intake were identified, both for total sodium intake (Model 2: p for interaction = 0.042) and fermented food-derived sodium intake (Model 3: p for interaction = 0.040), indicating that the association varied according to sodium intake levels. As shown in Figure 3, The difference in predicted probability of hs-CRP ≥1 mg/L by sodium intake level (ΔP) was more pronounced in the ≥65 year group when fermented food-derived sodium was considered. This pattern was not observed in adults aged 20–39 years and was less consistent in those aged 40–64 years.

## 4. Discussion

This study examined the association between fermented food consumption and hs-CRP across age groups in Korean adults, and investigated the moderating role of sodium intake in this relationship. The present findings suggest that the association between fermented food consumption and systemic inflammation is not uniform across age groups, and that sodium intake may serve as a contextual modifier of this relationship, particularly in older adults.

In the present study, socioeconomic status, lifestyle behaviors, and chronic disease prevalence all differed markedly across age groups. Physical activity, household income, and educational attainment declined significantly with advancing age, while the prevalence of chronic diseases and hs-CRP levels increased correspondingly. In addition, macronutrient intake also varied by age, with overall decreases in energy, protein, fat, and sodium intake and a relative increase in carbohydrate intake with advancing age. This pattern is consistent with the concept of inflammaging, characterized by a progressive increase in chronic low-grade systemic inflammation accompanying the aging process [27], which has been discussed as a pathophysiological substrate for age-related conditions including central obesity, insulin resistance, cardiovascular metabolic disease, and increased mortality. The significant age-related differences in hs-CRP observed in this study support the need to interpret the association between dietary factors and inflammation within an age-specific context, and suggest that the inflammatory implications of equivalent dietary exposures may differ by age.

Across age groups, fermented food intake ranged from approximately 262 to 301 g/day, contributing 7.4% to 10.1% of total energy intake. This estimate differed somewhat from those reported in prior studies. A study conducted among Japanese adults, which distinguished between fully fermented foods and partially fermented foods, reported a per capita intake of 438 g/d, corresponding to approximately 18% of energy intake [28], while a Dutch study analyzing data from the European Prospective Investigation into Cancer and Nutrition (EPIC-Netherlands) cohort, which restricted the definition to bacterially fermented foods, reported a fermented food intake proportion of 6.4% and a median intake of 128 g/d [29]. A more recent Swiss study estimated fermented food intake using criteria based on viable microorganism levels and fermented ingredients [30]. Differences in estimates across countries reflect not only variation in dietary cultures, but also the heterogeneity of definitions and classification criteria adopted across studies. Despite growing efforts to establish foundational data for fermented food intake guidelines across diverse national contexts, inconsistency in the definition and classification of fermented foods remains a persistent limitation. The development of internationally standardized criteria for defining fermented foods, alongside age-stratified analyses incorporating a broader range of dietary variables, would contribute to the formulation of more robust evidence-based dietary guidelines.

In this study, the proportion of total energy derived from fermented foods decreased significantly with advancing age, being highest in the 20–39-year group and lowest in the ≥65 year group. This finding contrasts with prior studies reporting higher fermented food consumption among older adults [31,32,33], which has been attributed to a stronger preference for home-prepared and traditional dietary patterns in this age group. In addition to generational preferences for traditional dietary patterns, socioeconomic and lifestyle factors may also contribute to these differences. Variations in income level, education, and food accessibility across age groups may influence food choices and the types of fermented foods consumed. When examined by food group, the distribution of fermented food intake revealed distinct age-related patterns. In the 20–39-year group, the energy contribution from fermented grain products, dairy, and alcoholic beverages was relatively high, whereas in older age groups, jang and vegetable-based fermented foods accounted for a greater proportion. This pattern is consistent with national nutrition survey data, which show higher consumption of bread and dairy products among younger adults and increasing reliance on kimchi and jang among older adults [34]. These age-related differences extend beyond intake quantity and reflect variations in nutrient composition and sodium content across food groups. Given that kimchi and jang, which predominate in the diets of older adults, are major dietary sources of sodium in the Korean diet, older adults may have higher sodium intake from fermented foods than younger individuals, even at comparable total intake levels. This highlights the importance of considering not only the quantity of fermented food consumption, but also the composition of food groups and accompanying sodium load when interpreting their health implications.

In this study, the association between fermented food intake and the risk of hs-CRP ≥1 mg/L differed across age groups. Among adults aged 20–39 and 40–64 years, higher fermented food intake was consistently associated with lower odds of elevated hs-CRP. This inverse relationship remained after adjustment for sodium intake, suggesting that the observed association is not solely explained by sodium. Previous randomized controlled trials and meta-analyses have reported inverse associations between fermented dairy consumption and CRP levels [13,35,36]. In the present study, fermented dairy products contributed a relatively larger proportion of total fermented food intake, particularly among younger adults, which may partly explain the observed association. In addition, fermentation-derived compounds, such as organic acids, bioactive peptides, and microbial metabolites, may influence gut microbiota composition and immune function, thereby contributing to reduced systemic inflammation [21,37].

In contrast, among adults aged ≥65 years, no overall association was observed between fermented food intake and hs-CRP. However, significant interactions with sodium intake were identified, indicating that the association differed depending on sodium intake levels. Further analyses showed that the inverse association between fermented food intake and hs-CRP was more evident at lower sodium intake levels and became attenuated as sodium intake increased. This pattern was not observed in the younger age groups. These findings are consistent with previous research suggesting that sensitivity to sodium increases with age, possibly due to reduced renal function, changes in vascular responsiveness, and alterations in immune regulation [8,9]. Such age-related differences may help explain why sodium plays a more prominent role in shaping the relationship between fermented food intake and inflammation in older adults.

These findings suggest that when evaluating the health effects of fermented foods in older adults, it is necessary to consider sodium content within a broader dietary pattern framework, rather than treating fermented food consumption as an isolated dietary factor. Given that the anti-inflammatory effect of fermented foods appeared more evident under lower sodium conditions, dietary strategies that prioritize low-sodium fermented food options or concurrent reductions in sodium intake may represent a viable approach to managing chronic inflammation in older adults.

This study has several limitations. First, the cross-sectional design precludes causal inference between fermented food consumption and hs-CRP. Second, dietary intake was assessed using a single 24 h recall, which may not reflect usual intake. Third, fermented food-derived sodium was estimated using a food composition database, and variability in actual sodium content may not have been fully captured. Fourth, fermented foods were not sub-classified into traditional Korean fermented foods and non-traditional fermented foods. Given their substantial differences in sodium content and microbial composition, their differential associations with systemic inflammation may not have been fully captured. Finally, residual confounding cannot be excluded, and the findings may not be generalizable beyond Korean adults. Despite these limitations, this study provides evidence on age-specific associations between fermented food intake and systemic inflammation and highlights the potential moderating role of sodium.

## 5. Conclusions

This study systematically analyzed the association between fermented food consumption and systemic inflammatory markers across age groups in Korean adults, and examined the moderating role of sodium intake in this relationship. Age-stratified analyses revealed that the association between fermented food consumption and hs-CRP differed by age group, and that sodium intake level significantly moderated this association in the 65 years and older group. These findings suggest that the health implications of fermented food consumption should be evaluated in relation not only to total intake, but also to the specific types of fermented foods consumed and their accompanying sodium burden, particularly in older adults.

## Figures and Tables

**Table 1:** General characteristics by age.

Variables	20–39 y (*n* = 4410)		40–64 y (*n* = 8742)		≥65 y (*n* = 4832)		*p*-value
Sex							<0.001
Men	1989	(45.1)	3627	(41.5)	2115	(43.8)	
Women	2421	(54.9)	5115	(58.5)	2717	(56.2)	
Age (year)	30.02	±0.130	51.24	±0.101	72.54	±0.091	<0.001
BMI (kg/m^2^)	24.06	±0.066	24.35	±0.041	24.37	±0.049	<0.001
WC (cm)	81.47	±0.185	83.42	±0.124	86.07	±0.159	<0.001
Household income							<0.001
Lowest	308	(7.0)	910	(10.4)	2218	(46.2)	
Lowest middle	1079	(24.5)	1991	(22.8)	1333	(27.7)	
Upper middle	1546	(35.1)	2585	(29.6)	751	(15.6)	
Highest	1468	(33.4)	3233	(37.1)	502	(10.4)	
Education status							<0.001
Elementary school or less	25	(0.6)	1079	(13.0)	2661	(58.1)	
Middle school	72	(1.7)	1067	(12.9)	678	(14.8)	
High school	1480	(34.9)	3087	(37.3)	797	(17.4)	
College or higher	2659	(62.8)	3050	(36.8)	442	(9.7)	
Marital status, yes	3332	(15.8)	11,261	(53.5)	6458	(30.7)	<0.001
Alcohol consumption, yes	3773	(85.7)	6528	(74.8)	2451	(50.9)	<0.001
Current smoking, yes	1670	(37.9)	3426	(39.3)	1795	(37.3)	0.055
Aerobic physical activity, yes	2332	(55.1)	3673	(44.4)	1490	(32.6)	<0.001
Hypertension, yes	77	(1.7)	1817	(20.8)	2693	(55.7)	<0.001
Diabetes, yes	23	(0.5)	676	(7.7)	1062	(22.0)	<0.001
Cardiovascular disease, yes	2	(0.0)	256	(2.9)	623	(12.9)	<0.001
Cancer diagnosis, yes	26	(0.6)	347	(4.0)	342	(7.1)	<0.001
hs-CRP (mg/L)	0.265	±0.008	0.268	±0.005	0.330	±0.008	<0.001

Values are presented as mean ± standard error (SE) for continuous variables and number (weighted percentage) for categorical variables. *p*-values were obtained from overall comparisons across age groups; no pairwise comparisons were conducted. Cardiovascular disease was defined as having been diagnosed with at least one of the following: angina pectoris, myocardial infarction, or stroke. Cancer diagnosis was defined as having been diagnosed with at least one of the following: gastric, liver, colorectal, breast, cervical, lung, or thyroid cancer. BMI, body mass index; WC, waist circumference; hs-CRP, high-sensitivity *C*-reactive protein.

**Table 2:** (**A**) Nutrient intake by age group; (**B**) Fermented food intake ratio and sodium intake derived from fermented food by age group and food category.

(**A**)															
Variables		20–39 y				40–64 y				≥65 y			*p*-value		
		Mean ± SE				Mean ± SE				Mean ± SE					
Energy intake (kcal)		2177.0		±15.3		2044.8		±10.3		1701.4		±11.6	<0.001		
Carbohydrates (g)		299.9		±2.1		308.9		±1.5		294.9		±1.98	<0.001		
Protein (g)		81.1		±0.7		72.3		±0.4		56.2		±0.48	<0.001		
Fat (g)		58.1		±0.6		43.5		±0.4		27.3		±0.36	<0.001		
Dietary fiber (g)		21.9		±0.2		27.2		±0.2		25.6		±0.25	<0.001		
Sodium (mg)		3839.8		±38.4		3703.9		±27.8		2958.2		±34.0	<0.001		
(**B**)															
Variables	20–39 y					40–64 y				≥65 y				*p*- value *	*p*- value †
	mean		±SE		Sodium intake (mg)	mean	±SE		Sodium intake (mg)	mean	±SE		Sodium intake (mg)		
Energy intake from fermented foods (%)	10.180		±0.183		1588.3	9.126	±0.114		1823.6	7.180	±0.126		1682.8	<0.001	<0.001
Grains (%)	3.845		±0.154		118.5	2.250	±0.081		53.7	1.544	±0.092		27.5	<0.001	<0.001
Jang (%)	1.500		±0.034		786.0	1.782	±0.024		866.5	1.808	±0.036		796.4	<0.001	<0.001
Vinegars (%)	0.006		±0.002		0.0	0.012	±0.004		0.2	0.004	±0.001		0.0	0.194	0.294
Vegetables (%)	1.395		±0.026		611.7	1.960	±0.023		807.5	1.990	±0.035		742.7	<0.001	<0.001
Fish (%)	0.050		±0.006		39.6	0.127	±0.010		74.5	0.120	±0.010		81.3	<0.001	<0.001
Fruits (%)	0.014		±0.002		4.4	0.006	±0.001		6.3	0.003	±0.001		10.3	<0.001	0.073
Dairy (%)	1.338		±0.065		26.0	1.051	±0.037		16.9	0.955	±0.050		11.8	<0.001	<0.001
Alcoholic beverages (%)	1.936		±0.094		3.1	1.907	±0.075		3.1	0.750	±0.063		0.9	<0.001	<0.001
Sauces (%)	0.020		±0.006		3.1	0.019	±0.005		1.7	0.004	±0.002		0.3	0.076	0.001
Teas·beverages (%)	0.077		±0.013		0.2	0.010	±0.004		0.0	0.003	±0.002		0.0	<0.001	<0.001

Values for fermented food intake are expressed as mean ± SE (% of total energy). Sodium intake derived from fermented food is expressed in mg/day. All variables were adjusted for total energy intake using the energy-adjustment method. * *p*-value for differences in fermented food intake ratio across age groups. † *p*-value for differences in sodium intake derived from fermented food across age groups.

**Table 3:** Age-specific odds of hs-CRP ≥1 mg/L according to fermented food intake.

Age Group	Q1		Q2	Q3	Q4	*p*-Value	p for Trend	p for Interaction †
20–39 y								
Model ^(1)^	1	ref.	1.029 (0.828–1.278)	0.843 (0.671–1.058)	0.708 (0.558–0.898)	0.007	0.001	
Model ^(2)^	1	ref.	1.025 (0.824–1.275)	0.839 (0.667–1.055)	0.706 (0.556–0.898)	0.007	0.001	0.302
Model ^(3)^	1	ref.	1.018 (0.814–1.274)	0.832 (0.654–1.058)	0.699 (0.542–0.901)	0.008	0.002	0.715
40–64 y								
Model ^(1)^	1	ref.	0.976 (0.832–1.144)	0.846 (0.715–1.001)	0.846 (0.721–0.993)	0.069	0.013	
Model ^(2)^	1	ref.	0.970 (0.826–1.139)	0.839 (0.707–0.996)	0.841 (0.716–0.989)	0.062	0.012	0.048
Model ^(3)^	1	ref.	0.948 (0.804–1.117)	0.812 (0.672–0.972)	0.817 (0.691–0.967)	0.036	0.006	0.459
≥65 y								
Model ^(1)^	1	ref.	0.842 (0.682–1.040)	1.056 (0.861–1.295)	0.907 (0.742–1.109)	0.120	0.836	
Model ^(2)^	1	ref.	0.828 (0.668–1.026)	1.024 (0.830–1.264)	0.880 (0.713–1.086)	0.120	0.605	0.042
Model ^(3)^	1	ref.	0.831 (0.668–1.033)	1.031 (0.829–1.283)	0.882 (0.703–1.108)	0.118	0.653	0.040

WC, waist circumference. Values are odds ratios (ORs) with 95% confidence intervals (CIs). Q1 (lowest quartile of fermented food intake) was used as the reference group. Model 1 was adjusted for sex, age, cardiovascular disease, cancer diagnosis, WC, and total energy intake. Model 2 was additionally adjusted for sodium intake (g). Model 3 was additionally adjusted for sodium intake (g) from fermented food. † p for interaction represents the interaction between fermented food intake and sodium intake within each age group.

## Data Availability

The KNHANES database is publicly available at the KNHANES website accessed on 19 December 2024 (https://knhanes.kdca.go.kr/knhanes/main.do).

## Supplementary Materials

The following supporting information can be downloaded at https://www.mdpi.com/article/10.3390/nu18081264/s1. Table S1: Fermented Food Groups and Items. Table S2: Fermented foods intake by age.

## Abbreviations

hs-CRP	High-sensitivity *C*-reactive protein
KNHANES	Korea National Health and Nutrition Examination Survey
KDCA	Korea Disease Control and Prevention Agency
BMI	Body Mass Index
WC	Waist Circumference
AHA/CDC	American Heart Association/Centers for Disease Control and Prevention
ORs	Odds Ratios
CIs	Confidence Intervals
SE	Standard Error
