# Supplementary material for: The Association Between Fermented Food Intake and Hs-CRP Across Age Groups in Korean Adults: Effect Modification by Sodium Intake
Source: Nutrients. 2026 Apr 16;18(8):1264. doi: 10.3390/nu18081264 (PMC13119084; doi:10.3390/nu18081264)
Supplement: Supplementary file 1 [file nutrients-18-01264-s001.zip › nutrients-4226722-supplementary.pdf]

Table S1. Fermented Food Groups and Items

Food Groups	Food Items
Grains	baguette, sourdough bread, steamed bun, bagel, steamed fermented rice cake, naan, sikhye (korean sweet rice beverage), jocheong (korean rice syrup)
Fermented Soybean Products	ganjang (soy sauce), doenjang (fermented soybean paste), gochujang (fermented red pepper soybean paste), cheonggukjang (fast-fermented soybean paste), natto (Japanese fermented whole soybean paste), ssamjang (a mixture of gochujang and doenjang), chunjang (fermented black soybean paste)
Vinegar	vinegar, vinegar-based beverage, fruit vinegar
Vegetables	danmuji (yellow pickled radish), jangajji (korean pickled vegetables), kimchi
Fish and Seafood	jeotgal (fermented seafood), aekjeot (fermented fish sauce), fermented fish
Fruits	pickled plums, pickled olives
Dairy Products	liquid yogurt, yogurt, cheese
Alcoholic Beverages	beer, wine, makgeolli (korean rice wine), fruit wine, cheongju (korean refined rice wine)
Sauces	balsamic dressing, hot sauce, fish sauce
Leaf Teas and Beverages	fermented tea, fermented beverage

Table S2. Fermented foods intake by age

Variables	20-39 y		40-64 y		≥ 65 y		p value
Energy intake from fermented foods (g)	271.643	±5.711	301.342	±4.707	261.878	±4.320	<0.001
Grains (g)	33.064	±1.529	20.189	±0.896	16.597	±0.928	<0.001
Jang (g)	19.198	±0.459	23.156	±0.351	23.900	±0.494	<0.001
Vinegars (g)	1.338	±0.147	1.207	±0.095	0.881	±0.079	<0.001
Vegetables (g)	93.905	±1.855	132.552	±1.712	137.596	±2.488	<0.001
Fish (g)	0.995	±0.127	3.058	±0.362	2.565	±0.195	<0.001
Fruits (g)	0.223	±0.034	0.137	±0.024	0.162	±0.037	0.057
Dairy (g)	22.411	±1.184	22.481	±0.917	19.379	±1.209	<0.001
Alcoholic beverages (g)	92.896	±5.383	96.744	±4.546	59.556	±3.593	<0.001
Sauces (g)	0.224	±0.053	0.135	±0.054	0.075	±0.031	0.102
Teas · beverages (g)	7.391	±1.029	1.683	±0.316	1.168	±0.471	0.152

All nutrients and fermented food intake variables presented in this table were adjusted for total energy intake using the energy-adjustment method.
